# PDX1, Neurogenin-3, and MAFA: critical transcription regulators for beta cell development and regeneration

**DOI:** 10.1186/s13287-017-0694-z

**Published:** 2017-11-02

**Authors:** Yaxi Zhu, Qian Liu, Zhiguang Zhou, Yasuhiro Ikeda

**Affiliations:** 1 Department of Molecular Medicine, Mayo Clinic, College of Medicine, 200 First St. SW, Rochester, MN 55905 USA; 2 Department of Orthopedics, The Second Xiangya Hospital, Central South University, 139 Middle Renmin Road, Changsha, Hunan Province 410013 China; 3 Institute of Metabolism and Endocrinology, The Second Xiangya Hospital, Key Laboratory of Diabetes Immunology, Ministry of Education, Central South University, National Clinical Research Center for Metabolic Diseases, 139 Middle Renmin Road, Changsha, Hunan Province 410013 China; 4 Center for Regenerative Medicine, Mayo Clinic, College of Medicine, 200 First St. SW, Rochester, MN 55905 USA

**Keywords:** Diabetes, Trans-differentiation, Induced pluripotent stem cells, Embryonic stem cells, Regenerative medicine

## Abstract

Transcription factors regulate gene expression through binding to specific enhancer sequences. Pancreas/duodenum homeobox protein 1 (PDX1), Neurogenin-3 (NEUROG3), and V-maf musculoaponeurotic fibrosarcoma oncogene homolog A (MAFA) are transcription factors critical for beta cell development and maturation. NEUROG3 is expressed in endocrine progenitor cells and controls islet differentiation and regeneration. PDX1 is essential for the development of pancreatic exocrine and endocrine cells including beta cells. PDX1 also binds to the regulatory elements and increases insulin gene transcription. Likewise, MAFA binds to the enhancer/promoter region of the insulin gene and drives insulin expression in response to glucose. In addition to those natural roles in beta cell development and maturation, ectopic expression of PDX1, NEUROG3, and/or MAFA has been successfully used to reprogram various cell types into insulin-producing cells in vitro and in vivo, such as pancreatic exocrine cells, hepatocytes, and pluripotent stem cells. Here, we review biological properties of PDX1, NEUROG3, and MAFA, and their applications and limitations for beta cell regenerative approaches. The primary source literature for this review was acquired using a PubMed search for articles published between 1990 and 2017. Search terms include diabetes, insulin, trans-differentiation, stem cells, and regenerative medicine.

## Background

Type 1 diabetes mellitus (T1D) is an autoimmune-mediated disease in which pancreatic beta cells are destroyed by the immune system, thus causing life-long dependence on exogenous insulin therapy [1]. This can lead to hypoglycemic events, injection site complications, insulin resistance, and allergies along with several other issues, while a subset of patients experience difficulty in controlling fluctuations of blood glucose levels. Islet transplantation has emerged as a promising treatment option which helps the restoration of glycemic control and protection from severe hypoglycemic events. However, its widespread use has been limited due to insufficient donor resources, transplant rejection, and the necessity for lifelong immune suppression.

Transcription factors play critical roles in regulating gene expression. Ectopic expression of selected transcription factors can change, or trans-differentiate, the fate of somatic cells. This process is called cellular reprogramming. Reprogramming nonbeta cells into insulin-producing cells potentially offers novel regenerative approaches for T1D therapy. There are several transcription factors involved in early pancreatic progenitor formation, including pancreas/duodenum homeobox protein 1 (PDX1), forkhead box A2 (FOXA2), and sex determining region Y-box 17 (SOX17). Some transcription factors are critical for endocrine lineage specification and differentiation, such as Neurogenin 3 (NEUROG3) and neurogenic differentiation 1 (NEUROD1). Late maturation of beta cells is shown to be regulated by factors including V-maf musculoaponeurotic fibrosarcoma oncogene homolog A (MAFA), V-maf musculoaponeurotic fibrosarcoma oncogene homolog B (MAFB), paired box gene 6 (PAX6), and estrogen-related receptor gamma [2, 3]. Among these transcription factors, PDX1, NEUROG3, and MAFA are the most extensively explored. They have been repeatedly reported to be able to trans-differentiate various types of cells into insulin-positive cells, including pancreatic exocrine cells, hepatocytes, intestine cells, gall bladder cells, and stem cells. Here, we focus on these three key beta cell transcription factors and assess their applications for trans-differentiation of nonbeta cell types into insulin-producing cells.

## Biological properties of PDX1, NEUROG3, and MAFA

### PDX1

PDX1, also known as insulin promoter factor 1, is a homeodomain transcription factor. PDX1 expression is observed as early as embryonic day 8.5 (E8.5) at the 5–6 somite stages in mouse [4] and around gestational week 4 in human [5]. PDX1 is required for early embryonic development of the pancreas, as in human study a case report has shown a 5-year-old female Caucasian suffering from pancreatic agenesis because of a homozygous, single nucleotide deletion within the *PDX1* gene [6]. PDX1 is also required for the subsequent differentiation of pancreatic lineages. When the expression of PDX1 from E11.5 (after the formation of normal pancreatic epithelium and ductules) is blocked throughout the parturition in pregnant mice, the pancreatic development is also arrested as evidenced by the undeveloped pancreatic remnant consisting of only ducts but no acinar or beta cells [7]. In mature beta cells, depletion and reduction of PDX1 induces glucose intolerance, which suggests the critical role of PDX1 in maintaining beta cell function [7]. This notion is also supported by the identification of maturity-onset diabetes of the young 4 (MODY4), one type of diabetes caused by monogenic defects (heterozygous) in the *PDX1* gene. In nonobese diabetic (NOD) mice and human T1D patients, PDX1 autoantibodies are detected, suggesting PDX1 could be an autoantigen for T1D [8]. In human type 2 diabetes mellitus (T2D), PDX1 expression levels of islet beta cells are also compromised [9]. These data highlight the crucial roles of PDX1 in early embryonic pancreatic formation, specification of different endocrine lineages, and later maturation of beta cell function.

### NEUROG3

NEUROG3 is a member of the basic helix–loop–helix transcription factor family involved in the central nervous system and embryonic pancreas development. During the embryonic development of mouse pancreas, expression of NEUROG3 is first observed in the dorsal pancreatic epithelium at E9, increases between E9.5 and E15.5, and then decreases to a very low level in neonatal pancreas [10]. In human pancreatic development, the expression is seen from week 8 and reaches its peak at around week 11 [5]. NEUROG3 is regarded as the proendocrine gene critical for pancreatic endocrine fates since it does not coexpress with mature endocrine cell hormones including insulin, glucagon, somatostatin, and pancreatic polypeptide [11]. Forced expression of *Neurog3* gene in pancreatic precursor cells in mouse embryos, under the control of *Pdx1* promoter, gives rise to endocrine cell differentiation, primarily alpha cells, and blocks exocrine development. Conversely, in NEUROG3-deficient mice, four islet cell types (alpha, beta, delta, and pancreatic polypeptide cells) and endocrine precursor cells are not generated, and neonates die postnatally from diabetes [11]. Intriguingly, *Neurog3* ^+/−^ heterozygous islets show no notable difference from *Neurog3* ^+/+^ islets in expression of PDX1, NKX6.1, GLUT2, MAFA, and MAFB on a per cell basis [12], suggesting that as long as cells adopt an endocrine fate instead of an exocrine fate under NEUROG3 stimulation, a relatively low NEUROG3 level per cell does not significantly affect beta cell differentiation.

Despite these observations, it remains unclear whether NEUROG3 is absolutely required for beta cell development in humans. In contrast to mouse studies, all reported patients with biallelic mutations in *NEUROG3* have functional endocrine cells capable of releasing C-peptide despite severe enteric anendocrinosis from childhood [13]. All of these cases indicate the presence of insulin-secreting cells, and the reason for this is still elusive. It is possible that these mutations are hypomorphic or null, given the fact that functionality tests are mainly limited to their abilities to activate NEUROD1. Nonetheless, it is evident that NEUROG3 is of great importance for beta cell development and function as all biallelic mutated patients present with permanent diabetes, although the threshold level of NEUROG3 requirement may be relatively low since all heterozygous parents are not diabetic. In T1D *db/db* mice, the *NEUROG3* expression level is increased markedly [14]. In contrast, in human T2D beta cells, no evidence shows altered expression of *NEUROG3* [9]. In NOD mice, chronic pancreatic immune cell infiltration is correlated with the emergence of NEUROG3-positive cells, indicating some extent of beta cell neogenesis under autoimmune inflammation [15]. Similar to human T2D beta cells, a human study shows no difference in the percentage of NEUROG3 cells (5–10%) in healthy and T1D human islets [16].

### MAFA

MAFA, also known as RIPE3b1, is member of the MAF family of basic leucine zipper. It is identified as a transcription factor that specifically binds to a conserved insulin enhancer element RIPE3b/C1-A2 and activates insulin gene expression. In mice, MAFA is initially detected at E13.5 only in insulin-producing cells, and expresses exclusively in beta cells in the adult pancreas [17]. Similar to rodents, nearly no MAFA is detected in human embryo until week 21, and the expression gradually increases after birth [18]. The link between MAFA expression and GSIS development, and, further, between MAFA expression and the maturity of beta cells, is supported by studies in MAFA-deficient mice, which demonstrate impaired GSIS, abnormal architecture of islets, and overt diabetes by postnatal 50 weeks [19]. On the contrary, glucose responsiveness can be acquired in P2 neonatal islets if the MAFA expression is induced by adenoviral-mediated overexpression [20]. Human studies have revealed that T2D islets display poorer GSIS properties than normal islets, which are accompanied by compromised MAFA expression levels [9]. Additionally, functional polymorphisms of MAFA were shown to associate with T1D in the NOD mouse model and patients [21, 22]. For regenerative approaches, induction of MAFA expression is also important to regenerate functional and mature beta cells from pluripotent stem cells. Physiologically driven induction of MAFA in hESCs is shown to be beneficial in improving GSIS [23]. Also, ectopic expression of MAFA out of its normal developmental context at PDX1-positive pancreatic progenitors or NEUROG3-positive pancreatic endocrine progenitors is detrimental for beta cell differentiation [24], highlighting that the finely-tuned time window and expression levels of MAFA are crucial for proper beta cell maturation.

### Interaction among PDX1, NEUROG3, and MAFA

**Fig. 1:**
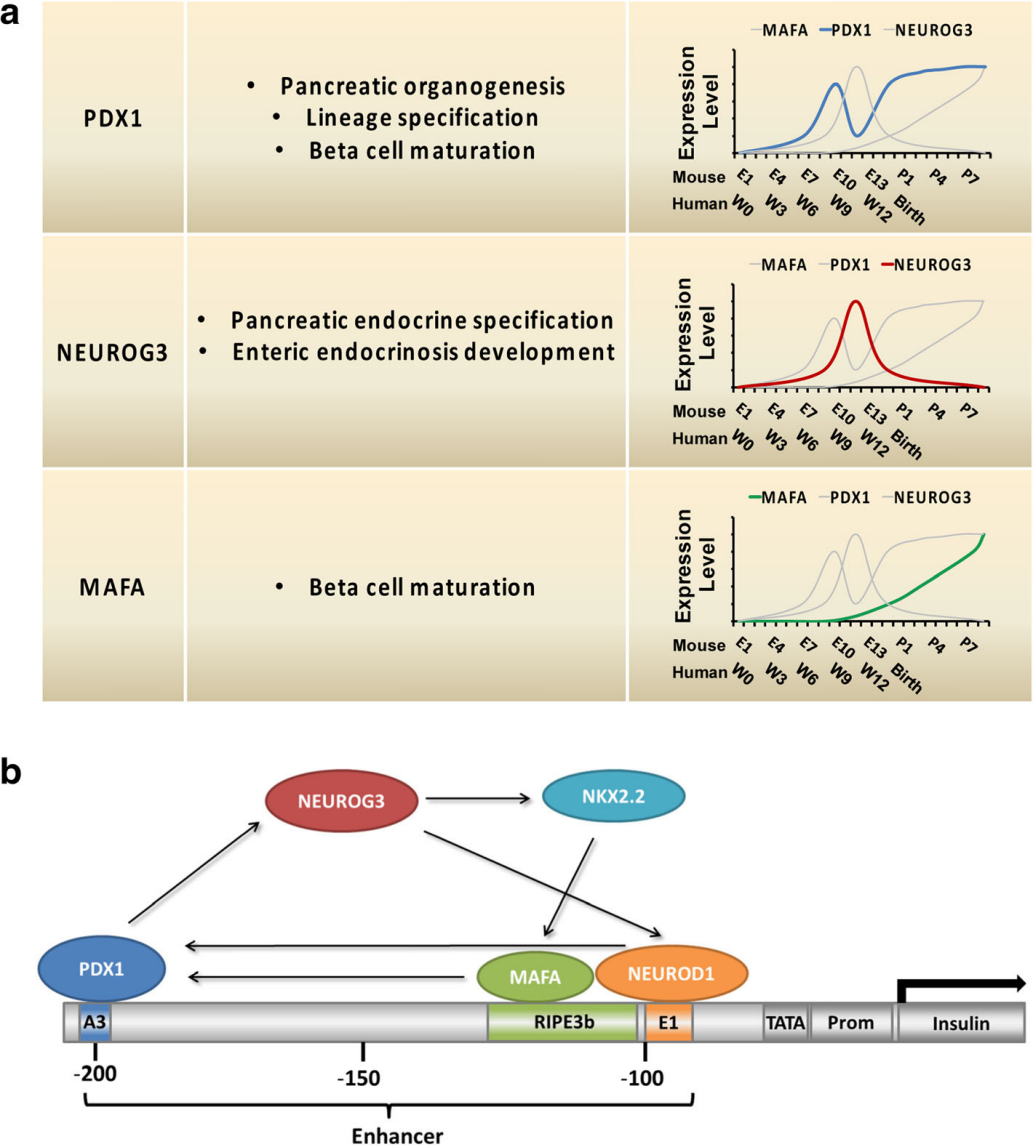
**a** Summary of primary endocrine function and expression patterns of PDX1, NEUROG3, and MAFA during mouse and human embryonic development. **b** Schematic of interaction of PDX1, NEUROG3, and MAFA in the activation of mouse insulin promoter. E1–E13 mouse embryonic day 1 to day 13, P1–P7 mouse postnatal day 1 to day 7, W0–W12 gestational week 1 to week 12, MAFA V-maf musculoaponeurotic fibrosarcoma oncogene homolog A, NEUROG3 Neurogenin 3, PDX1 pancreas/duodenum homeobox protein 1

In addition to their independent roles in beta cell development and maturation, PDX1, NEUROG3, and MAFA are also mutually interacted/regulated during the pancreatic developmental process. Because of the limited availability of human beta cells, the interaction among the three factors is better understood in rodent models. PDX1 regulates the expression of NEUROG3 [25]. Mice with homozygous *Pdx1* premature truncation mutation in the C terminus, which is dispensable for pancreas organogenesis, demonstrate a reduced number of NEUROG3-positive cells from E13.5 to P1. Additionally, transcript levels of *Neurog3* as well as other transcription factors regulating *Neurog3* gene expression, including *Sox9*, *Hnf6*, *Hnf1b*, and *Foxa2*, decrease in *Pdx1* mutated mice, suggesting that PDX1 regulates NEUROG3 directly but not solely through its role in the formation of pancreatic progenitor cells. PDX1 also regulates the expression of MAFA [26], since *Mafa* gene expression in *Pdx1* knockout mice is downregulated in islets compared with wildtype mice. NEUROG3 appears to have minimal effects on PDX1, as NEUROG3 disruption in hESCs only marginally reduced PDX1-positive cells compared with wildtype hESCs [27]. Other beta cell factors are also involved in the functional interactions between PDX1, NEUROG3, and MAFA. For instance, PDX1 directly interacts with NEUROD1 and forms a transcriptional activation complex on the insulin promoter [28]. In collaboration with NKX2.2, a downstream transcription factor of NEUROG3 [29], and FOXA2, a key marker gene for definitive endoderm cells, PDX1 regulates beta cell-specific MAFA expression through binding to the MAFA enhancer region [30]. MAFA and related MAFB proteins also regulate beta-cell-enriched PDX1 expression through binding to the Area II control region that contributes to PDX1 transcription in vivo [31] (Fig. 1).

## PDX1, NEUROG3, and/or MAFA facilitate trans-differentiation of nonbeta cells into insulin-producing cells

### Direct reprogramming to insulin-producing cells by ectopic expression of PDX1, NEUROG3, and/or MAFA

Reprogramming adult somatic cells into therapeutic cell types is an attractive approach for novel cell-based therapies for degenerative diseases. Recently, transcription factor-mediated “reprogramming”, or “trans-differentiation”, approaches have been used to regenerate beta-like cells or insulin-producing cells from nonbeta cell sources in vitro and in vivo. Notably, studies have demonstrated that overexpression of combinations of the three transcription factors, PDX1, NEUROG3, and/or MAFA, can reprogram the fate of various nonbeta cell types into insulin-producing cells. In the following paragraphs, we will summarize the progress of this unique approach.

### Background of trans-differentiation by single or dual beta cell factors

One of the first proof-of-concept studies was performed by Ferber et al., which demonstrates PDX1-mediated induction of insulin genes in the liver and amelioration of drug-induced hyperglycemia in diabetic mice upon hepatic PDX1 overexpression by an adenoviral vector [32]. NEUROG3 also displays efficacy in trans-differentiating hepatic cells into insulin-secreting cells which show a rapid but not sustained diabetes-reversal effect [33]. Subsequent studies attempted to further improve hepatic trans-differentiation by evaluating dual or more beta cell factors. In a study aiming to test different combinations of transcription factors for islet cell differentiation in the liver, PDX1 + NEUROD1, NEUROD1 + MAFA, and NEUROG3 + MAFA all demonstrated insulin gene activation, while the highest gene enrichment was gained by the combinatorial expression of all three factors. Consistently, NEUROG3 alone or NEUROG3 + MAFA can convert acinar cells to delta-like and alpha-like cells respectively, while only PDX1 + NEUROG3 + MAFA works synergistically to regenerate cells with beta cell features [34].

### Trans-differentiation of exocrine cells by PDX1, NEUROG3, and MAFA

Screenings of different combinations of critical transcription factors expressed in beta cells or their precursors in vivo, including NKX2.2, NKX6.1, PAX4, PAX6, NEUROG3, NEUROD1, PDX1, ISL1, and MAFA, have identified the combination of PDX1, NEUROG3, and MAFA (PNM) as a critical reprogramming factor to trans-differentiate pancreatic exocrine cells into insulin-producing beta-like cells [35]. Overexpression of PNM by adenoviral vectors is necessary and sufficient to trans-differentiate pancreatic exocrine cells into beta-like cells both morphologically and functionally, although derived beta-like cells do not form islet-like structures. Similar results have been reported in human exocrine pancreas reprogrammed by PNM plus PAX4 [36].

### PNM-mediated trans-differentiation of liver cells and other adult cell types

In addition to adult pancreatic exocrine cells, other adult cell types are also utilized for beta cell regeneration. Plasmid-based PNM gene delivery into the inferior vena cava segment has facilitated transient induction of insulin transcripts in rat livers [37]. Upon systemic administration of a single adenoviral vector encoding PNM factors in immunocompromised mice, Banga et al. demonstrated that duct-like SOX9-positive cells in the liver are directly reprogrammed into insulin-producing cells [38]. Notably, those insulin-producing duct cells have displayed some extent of glucose responsiveness ex vivo and the capacity of reversing experimental hyperglycemia in vivo. In immunocompetent mice, adenoviral vector-mediated PNM delivery has facilitated transiently induced insulin-producing SOX9-positive duct cells in the liver [39]. However, the conversion is not considered complete because induced insulin-producing cells are typically multihormone-positive and not glucose sensitive.

These observations clearly indicate the ability of the PNM cocktail for beta cell regeneration from liver cells. However, it does not rule out the contributions of generation of insulin-producing cells from nonliver cell types. To address this, Chen et al. performed an in-vivo screening study of a wide spectrum of tissues and found that duodenum and jejunum tissues are more “susceptible” to the PNM-mediated trans-differentiation than other tissues [40], although it is challenging to fully compare the regenerative efficiency among different tissues, especially pancreatic exocrine, liver, and intestinal tissues, because various factors (e.g., stoichiometry, gene delivery, and expression efficiency) can affect the regeneration efficiency. Although not fully functional, these neo-beta cells from the intestine still appear to have several advantages including the lack of glucagon or multihormonal expression and an abundance of cell sources [40]. Additionally, adenoviral-mediated PNM expression can also trans-differentiate primary mouse gall bladder epithelial cells into insulin-positive cells partially benefiting from a common developmental origin between extrahepatic biliary tissue and ventral pancreas [41].

### PNM-supported differentiation of pluripotent and adult stem cells

Pluripotent stem cells, such as hESCs and induced pluripotent stem cells (iPSCs), are characterized by pluripotency and infinite propagation. Their successful differentiation into insulin-producing progeny could provide unlimited cell sources for islet regeneration. For translational purposes, stem cells are a preferable cell source for beta cell regeneration compared with somatic cells, as adult somatic cell reprogramming appears to require developmentally closely related cell types (e.g., pancreas, liver, and intestine) for high reprogramming efficiency [40, 42]. Xu et al. assessed the capacity of mouse ESCs for beta cell differentiation upon overexpression of PNM and NEUROD1 at different stages of guided differentiation and with various combinations [43]. They demonstrated that coexpression of PNM showed significantly higher induction of the insulin gene when compared with two factors and a single factor strategy. Of note, when compared between PNM and PN + NeuroD1, PN + NeuroD1 transduction activated *Ins1* and *Ins2* gene expression better. However, somatostatin gene expression levels are much lower in PNM, suggesting that PNM-induced cells may be more lineage specific.

The major impediments in utilizing hESCs are ethical issues and immunological intolerance. Human iPSCs are ideal cell sources for beta cell regeneration given that they are similar to hESCs and free from ethic constraints associated with the use of embryo-derived cells. Saxena et al. have shown that human iPSC-derived pancreatic progenitor cells can be differentiated into glucose-sensitive beta-like cells using a synthetic lineage-control network to express PNM in a dynamic way, mimicking the intrinsic expressing timeline as introduced previously [44]. This preprogrammed sequential differentiation system is technically more advanced than simple PNM overexpression. However, the ability of the differentiated cells to rescue diabetes in an animal model remains to be determined. Encouragingly, there are several ongoing/planning phase clinical trials testing the safety and efficacy of stem-cell-derived cell products. A first in human, phase I/II clinical trial is being conducted by ViaCyte to test its stem-cell-derived pancreatic progenitor cells in suitable T1D patients [45]. Meanwhile, the Boston Autologous Islet Replacement Program (BAIRT) is planning a clinical trial to transplant autologous iPSC-derived beta-like cells into suitable candidates [46]. These clinical trials would provide important evidence for translating beta cell regeneration discoveries into cures for diabetes.

Based on the critical roles of PNM for beta cell development and function, pharmacological approaches to induce endogenous PNM genes, an alternative to exogenous genetic manipulations, have been studied extensively in terms of a therapeutic perspective. Growth factors/small molecules such as retinoid acid, fibroblast growth factor 7 and 10, sonic hedgehog signaling inhibitors (e.g., Sant-1), and protein kinase C signaling pathway activators (e.g., indolactam V) can upregulate PDX1 expression [47]. TGF-β type I receptor inhibitors (e.g., Alk5 inhibitor II), vesicular monoamine transporter 2 inhibitor, reserpine, and tetrabenazine can induce NEUROG3 expression [48]. MAFA transcript levels can be induced by a formula including AXL inhibitor (R428), *N*-acetyl cysteine, Alk5 inhibitor II, and thyroid hormone [23]. The application of these growth factors/small molecules has overcome several disadvantages of genetic modification, and provided a safe, efficient, and scalable approach for beta cell regeneration.

## Conclusion

Regenerative medicine has opened a new era for T1D cell therapy approaches, which may have greater effectiveness, safety, and versatility over exogenous insulin therapy. Key beta cell transcription factors, PDX1, NEUROG3, and/or MAFA, have been studied extensively for their roles in beta cell development and function, as well as their potential applications for trans-differentiating nonbeta cells into insulin-producing cells. However, it is of note that the PNM gene combination generally does not reprogram nonbeta cells into genuine beta cells, and that successful reprogramming into insulin-producing cells typically needs developmentally related cell types rather than unrelated types [42]. For successful clinical applications of the exciting “trans-differentiation” concept, we will likely require trans-differentiation into genuine beta cells, which demonstrate appropriate glucose and incretin responsiveness. We recently found that introduction of PDX1 in definitive endoderm, NEUROG3 in pancreatic endoderm, and MAFA in pancreatic endocrine progenitor cells during a stepwise differentiation process could accelerate regeneration of glucose-responsive and incretin (GLP-1)-responsive beta cells from human pluripotent stem cells in vitro (Zhu et al., manuscript in preparation). Further understanding of the underlying mechanism in the PNM-mediated reprogramming and careful optimization of PNM introduction conditions, such as the timing, duration, expression levels, and delivery strategies, would inform the rational design of next-generation PNM-mediated therapy for diabetes. Our iPSC differentiation system would provide a novel in-vitro model system to characterize the optimal timing, duration, level, and method of PNM introduction for mature beta cell regeneration.

## Abbreviations

GSIS: Glucose-stimulated insulin secretion
hESC: Human embryonic stem cell
iPSC: Induced pluripotent stem cell
MAFA: V-maf musculoaponeurotic fibrosarcoma oncogene homolog A
NEUROG3: Neurogenin 3
NOD: Nonobese diabetic
PDX1: Pancreas/duodenum homeobox protein 1
PNM: PDX1, NEUROG3, and MAFA
T1D: Type 1 diabetes mellitus
T2D: Type 2 diabetes mellitus
